# Correction: Vargas et al. Solving Schrödinger Bridges via Maximum Likelihood. *Entropy* 2021, *23*, 1134

**DOI:** 10.3390/e25020289

**Published:** 2023-02-03

**Authors:** Francisco Vargas, Pierre Thodoroff, Austen Lamacraft, Neil Lawrence

**Affiliations:** 1 The Computer Laboratory, Department of Computer Science and Technology, University of Cambridge, William Gates Building, 15 JJ Thomson Avenue, Cambridge CB3 0FD, UK; 2 The Cavendish Laboratory, Deparment of Physics, The Old Schools, Trinity Ln, Cambridge CB2 1TN, UK

In the original publication [1], there are two errors:

1. In the proof for observation 1 (Observation A1 in the appendix) there is a mistake due to a conflict of edits that significantly changed the proof. Rather than saying the KL goes to 0 which is incorrect we should say it goes to the ratio of marginals which coincides with the half bridge solution. A correction has been made to Appendix C. Proof Sketches for Half Bridges, Observation A1, Proof. (Sketch) W.l.o.g. More precisely, the proof should be:

**Proof.**  (Sketch) W.l.o.g., Consider the decomposition of the KL divergence that follows from the disintegration Theorem (Appendix B):

$$\begin{array}{cc} D_{\mathrm{KL}}\left(P||Q\right)= & D_{\mathrm{KL}}(\pi_{0}^{P}\left|\right|\pi_{0}^{Q})+E_{\pi_{0}^{P}}\left[D_{\mathrm{KL}}\left(P\left(\cdot |x\right)||Q\left(\cdot |x\right)\right)\right]. \end{array}$$

Furthermore, the disintegration Q(·|x(0)) is a solution to the dynamics $dx^{+}\left(t\right)=b^{+}\left(t\right)+\sqrt{\gamma }d\beta^{+}\left(t\right)$. We can make the term $E_{\pi_{0}^{P}}\left[D_{\mathrm{KL}}\left(P\left(\cdot |x\right)||Q\left(\cdot |x\right)\right)\right]$ go to 0 by setting P(·|x(0))=Q(·|x(0)), it is clear the dynamics of P(·|x(0)) follows $dx^{+}\left(t\right)=b^{+}\left(t\right)+\sqrt{\gamma }d\beta^{+}\left(t\right)$. What is left is to attach the constraint via $x\left(0\right)∼\pi_{0}\left(x\left(0\right)\right)$ which brings $D_{\mathrm{KL}}(\pi_{0}^{P}\left|\right|\pi_{0}^{Q})$ to $D_{\mathrm{KL}}(\pi_{0}\left|\right|\pi_{0}^{Q})=D_{\mathrm{KL}}(P^{*+}\left||Q\right)$ coinciding with the half bridge minima as per [20,22]. This is simply enforcing the constraints via the product rule (Disintegration Theorem) and then matching the remainder of the unconstrained interval with the disintegration for the reference distribution Q, following Equation (7). □

2. The following sentence of line following Equation (A45), should be deleted. As before, was included in the paper erroneously due to a conflict of edits, and the current wording is unclear. A correction has been made to Appendix D. Proof Sketch for Reverse-MLE Consistency, Lemma A3. (Convergence of discrete time reversal), Proof.

Finally note a potential proof strategy to extend Theorem 1 to EM Samples would be to exploit the strong convergence properties of the Euler scheme to show that the stochastic integral from Theorem 1 also converges in the case of Euler samples. This remains an interesting question for future work.

The authors state that the scientific conclusions are unaffected. This correction was approved by the Academic Editor.
